# Physiological Demands Across Exercise Intensity Domains in Rowing: Implications of Weight Category and Sex Differences

**DOI:** 10.3390/sports13080245

**Published:** 2025-07-25

**Authors:** Manoel Rios, Ricardo Cardoso, Ana Sofia Monteiro, João Paulo Vilas-Boas, Ricardo J. Fernandes

**Affiliations:** 1 Centre of Research, Education, Innovation and Intervention in Sport and Porto Biomechanics Laboratory, Faculty of Sport, University of Porto, 4200-450 Porto, Portugal; up201200394@up.pt (R.C.); sofiammonteirto@fade.up.pt (A.S.M.); jpvb@fade.up.pt (J.P.V.-B.); ricfer@fade.up.pt (R.J.F.); 2 Piaget Research Center for Ecological Human Development, Higher School of Sport and Education, Jean Piaget Polytechnic Institute of the North, 4405-678 Vila Nova de Gaia, Portugal

**Keywords:** oxygen uptake, lactate, weight category, gender differences, energy systems, ergometer

## Abstract

We examined the physiological demands of trained rowers across four exercise intensity domains (considering the effects of weight category and sex). Twenty-four trained rowers (12 lightweight and 12 heavyweight) performed 7 × 3 min incremental bouts on a Concept2 rowing ergometer (30 W power increases and 60 s rest intervals). Performance, cardiorespiratory and metabolic responses were continuously assessed throughout the experimental protocol to characterize internal load across progressive exercise intensities. Statistical analyses included a repeated measures ANOVA test and independent *t*-tests (*p* ≤ 0.05). Heavyweight rowers exhibited greater absolute anaerobic energy production in the severe domain (41.25 ± 10.39 vs. 32.54 ± 5.92 kJ) (*p* = 0.02), higher peak metabolic power (up to 1.57 ± 0.30 vs. 1.48 ± 0.30 kW) (*p* = 0.001) and greater total energy expenditure (up to 277.52 ± 51.23 vs. 266.69 ± 51.59 kJ) (*p* = 0.001) than lightweight rowers, whereas the latter showed comparable relative cardiorespiratory responses to heavyweights. With respect to sex differences, males demonstrated higher oxygen uptake (from ~43–59 vs. ~34–48 mL·kg^−1^·min^−1^) (*p* = 0.001), ventilation (from ~78–146 vs. ~49–99 L·min^−1^) (*p* = 0.001), metabolic power (from ~1.1–1.7 vs. ~0.7–1.0 kW) (*p* = 0.001) and energy expenditure (from ~193–305 vs. ~119–209 kJ) (*p* = 0.001) across all intensity domains. However, blood lactate levels and anaerobic energy contributions were similar between sexes. These findings demonstrated that domain-based physiological profiling effectively differentiates internal responses among rowers by weight category and sex. Heavyweights showed greater absolute energy output, while lightweights demonstrated higher metabolic efficiency. Males had elevated cardiorespiratory and metabolic values, but relative bioenergetic responses were similar across groups. These findings support individualized training based on physiological profiles.

## 1. Introduction

Rowing is a physiologically demanding discipline that requires the coordinated activation of large muscle groups in prolonged high-intensity sport events [1,2]. These efforts involve a complex interplay between the aerobic and anaerobic energy systems, which are activated differently depending on the intensity of the exercise [3,4]. Rowing performance depends not only on mechanical output and technical efficiency but also on internal physiological responses (e.g., oxygen uptake, ventilation and blood lactate accumulation). These variables vary systematically across exercise intensity domains (i.e., low, moderate, heavy and severe), each representing distinct physiological conditions [5,6,7]. Investigating exercise intensity domains provides a clearer understanding of the body’s physiological adaptations to increasing effort. While structured, domain-specific analyses under controlled laboratory conditions are well established in cycling and running [8,9]; rowing research has largely focused on maximal tests and time trials, with limited exploration of domain-specific physiological responses [7,10,11,12].

The classification of intensity into distinct physiological domains provides a valuable framework for evaluating internal load during exercise [9,13]. These domains are delineated based on metabolic thresholds (i.e., anaerobic threshold and maximal oxygen uptake), which reflect transitions in substrate utilization, fatigue onset and recovery demands [14]. Accurate identification and monitoring of these domains allow for targeted training prescriptions that align with individual physiological capacities. This is especially relevant in rowing, where improper intensity regulation can lead to suboptimal adaptation or overtraining [15,16]. Despite their potential, domain-specific analyses remain underutilized in applied rowing research, particularly for differentiating physiological responses across body mass classifications and between sexes [17].

Rowers are categorized into lightweight and heavyweight divisions to promote competitive equity, but this classification also results in distinct biomechanical and physiological responses [18]. Lightweight rowers generally exhibit lower absolute power outputs but often possess greater efficiency, prioritizing a high power-to-weight ratio and precise metabolic control, which help them sustain high intensities with less physiological strain compared with their heavyweight counterparts [19]. In contrast, heavyweight rowers tend to rely on greater muscle mass producing higher forces and power [20,21]. These differences affect how energy systems are engaged throughout efforts of different intensities and may alter the physiological cost of exercise at each domain. Understanding these variations is essential for optimizing training load and ensuring that conditioning programs reflect the specific energetic demands of each category [22,23].

Sex-based differences in strength and endurance capacities contribute to the physiological factors underlying variations in physical fitness between men and women. Males typically display higher values of oxygen uptake, cardiac output, hemoglobin concentration and ventilation, contributing to greater aerobic capacity and absolute performance potential [24,25]. In contrast, females often demonstrate different energy utilization profiles, favoring oxidative metabolism and presenting variations in lactate production and clearance [26,27]. These physiological differences influence the way internal load is managed across intensity domains and highlight the need for sex-specific considerations when designing training prescriptions. Despite their relevance, direct comparisons of physiological responses between male and female rowers across different exercise intensities remain limited in the scientific literature [24].

Technological advances in portable gas and lactate analyzers allow accurate, real-time assessments of physiological responses during exercise [7]. These tools offer detailed data on oxygen uptake, ventilation patterns, energy expenditure and the ratio between aerobic and anaerobic metabolism [24]. Although rowing performance is well known to depend on strength, power and anthropometric factors, relatively few studies have investigated physiological responses to incremental exercise across distinct intensity domains, and even fewer have simultaneously examined how these responses vary by sex and weight class [6,18]. For example, Penichet-Tomas et al. [6] assessed energy system contributions in elite male lightweight and heavyweight rowers and reported greater anaerobic involvement in heavyweights at high intensities, indicating distinct metabolic demands. Keenan et al. [18] analyzed performance trends over two decades and highlighted that sex-based differences in rowing performance are influenced not only by physiological factors but also by training opportunities and category-specific participation. Integrating domain-specific physiological data into training design may enhance the precision of intensity and volume prescriptions for the different groups. This approach helps detect early signs of maladaptation and supports individualized recovery strategies. In elite performance environments, small mismatches between physiological responses and external work can compromise adaptation.

The current study aimed to analyze the physiological responses of trained rowers from different weight categories and sexes across four exercise intensity domains during a standardized rowing ergometer protocol. The primary objective was to compare cardiorespiratory, metabolic and performance-related variables between lightweight and heavyweight rowers. The secondary objective was to examine differences between male and female rowers under the same exercise conditions. Based on the existing literature and physiological principles, we formulated the hypotheses that (i) heavyweight rowers would display higher cardiorespiratory responses and greater anaerobic energy contributions than lightweight rowers due to greater muscle mass and power output capacity; and (ii) male rowers would demonstrate higher metabolic power and oxygen transport variables compared with females, consistent with prior findings showing sex-based differences in aerobic capacity and muscle composition.

## 2. Materials and Methods

### 2.1. Study Design

Following a cross-sectional design, 24 trained national-level rowers volunteered to participate in the current study. All tests were conducted in a single-visit protocol, during the preparatory period of the season, in a controlled laboratory environment under consistent conditions (ambient temperature of 23 °C, 60% humidity and between 8:00 and 12:00 a.m.) and under the supervision of experienced researchers. Participants body mass, body fat percentage and fat-free mass were assessed using a bioelectrical impedance scale (InBody 230; InBody Co., Ltd., Seoul, Republic of Korea), while height was measured with a stadiometer (Seca 206; Seca GmbH, Hamburg, Germany). The experimental protocol performed on a rowing ergometer (Model D, Concept2, Morrisville, VT, USA) consisted of 7 × 3 min bouts with 30 W increments (starting at 180 and 120 W for the male and the female participants, respectively) and a 60 s passive rest interval between steps [24].

### 2.2. Participants

Participant demographics and anthropometric characteristics are displayed in Table 1. Participants were included in the study based on the following criteria: (i) a minimum of three years of experience in rowing training and competition; (ii) age between 18 and 40 years; and (iii) absence of musculoskeletal injuries within the past six months. Participants were excluded if they had any diagnosed cardiovascular, metabolic or respiratory conditions or were taking medications that could affect exercise performance. Prior to data collection, individuals were advised to abstain from intense physical exertion and to adhere to their regular dietary routines, avoiding the consumption of alcohol, caffeine and nutritional supplements during the 24 h preceding the assessment. Ethical approval for the study was granted by the institutional research ethics committee (CEFADE 27/2020), and all procedures complied with the Declaration of Helsinki. Participants were fully informed about the potential risks and benefits of the research and signed written consent forms before participation.

### 2.3. Procedure

Pulmonary gas exchange variables were continuously assessed on a breath-by-breath basis using a portable telemetric gas analyzer (COSMED K5; Cosmed, Rome, Italy), hung over the participant’s head to reduce interference during rowing. Heart rate was continuously recorded at baseline and during the test via a chest strap monitor (Garmin Edge 830; Garmin, Olathe, KS, USA), which transmitted data wirelessly to the K5 unit. To evaluate the contribution of the anaerobic system, capillary blood samples were collected from the earlobe (Lactate Pro2; Arkay, Inc., Kyoto, Japan) at rest, during the intervals and at the 1st, 3rd, 5th and 7th min of the recovery period until maximum lactate concentrations were detected [5].

The average values of the cardiorespiratory variables (e.g., oxygen uptake, respiratory frequency, ventilation, carbon dioxide, respiratory exchange ratio and heart rate) were calculated based on data collected during the final 30 s of exercise in each step [28]. The dataset was thoroughly assessed, and any breaths associated with coughing or signal disruptions were excluded [29]. Only values falling within the range of the mean ± 3 standard deviations were included in the subsequent analysis [5,29]. A smoothing technique was then applied, using a moving average over three breaths and a temporal average over 10 s. Standard physiological criteria were applied to determine maximal oxygen uptake, which included: (i) a plateau in its value between the final two stages (≤2.1 mL·kg^−1^·min^−1^); (ii) blood lactate concentration ≥8 mmol·L^−1^; (iii) respiratory quotient ≥1.0; (iv) heart rate > 90% of maximal; and (v) voluntary exhaustion (assessed visually and individually) [9]. The lactate–power curve was used to assess the anaerobic threshold through the determination of the interception point of the best fit of a combined linear and exponential pair of regressions [5], allowing determination of the following exercise intensity domains: (i) low and moderate intensities (corresponding to the two steps below and the anaerobic threshold, respectively); and (ii) heavy and severe intensities (matching the steps below and the step where maximal oxygen uptake was elicited, respectively) [5].

The energy expenditure was estimated as the sum of aerobic and anaerobic lactic energy contributions [10,30], with the former being calculated based on the time integral of net oxygen uptake (difference between each step mean value and the baseline value), while the latter was assessed based on the equation: $\mathrm{Anaerobic}\;\mathrm{lactic}\;=\;b\;[{\mathrm{La}}^{-}{]}_{\mathrm{net}}\;M$, where $[{\mathrm{La}}^{-}{]}_{\mathrm{net}}$ represents the difference between each step’s blood lactate concentrations and the baseline values, b denotes the constant for oxygen equivalent of the $[{\mathrm{La}}^{-}{]}_{\mathrm{net}}$ (= 3 mL·kg^−1^·mM^−1^) and *M* corresponds to the subject body mass (kg) [10,30]. Energy contributions from the different metabolic pathways are expressed in kJ, assuming an energy equivalent of 20.9 kJ·L^−1^ [31]. To estimate metabolic power, energy expenditure was divided by the total duration(s) of the exercise in each intensity step [10].

### 2.4. Statistical Analysis

An a priori analysis was conducted using G*Power software (version 3.1.9.7; Heinrich-Heine-Universität Düsseldorf, Düsseldorf, Germany) to determine the required sample size. Assuming a medium-to-large effect size (f = 0.50), consistent with previous work on incremental rowing and exercise testing, an alpha level of 0.05 and a power of 0.80, the analysis indicated that a total of 22 participants would be sufficient to detect a statistically significant effect [5,32]. All statistical analyses were performed using SPSS version 30.0 for Windows (IBM Corp., Armonk, NY, USA), the Shapiro–Wilk test assessed data normality and mean ± standard deviation values were calculated for all variables for each intensity domain. A one-way repeated measures ANOVA test was performed to determine the incremental behavior of the analyzed variables across the intensity domains, and multiple post hoc pairwise comparisons were conducted using Bonferroni correction to detect differences in the cardiorespiratory and metabolic variables between exercise intensities. The independent samples *t*-test was applied to compare lightweight and heavyweight rowers, as well as between sexes, cardiorespiratory and metabolic variables. The significance level was set at *p* ≤ 0.05 for all statistical analyses, and Cohen’s d effect size was computed to assess the magnitude of changes (trivial: <0.2; small: 0.2–0.6; moderate (0.6.1.2; large (1.2–2.0; and very large >2.0.

## 3. Results

**Figure 1:**
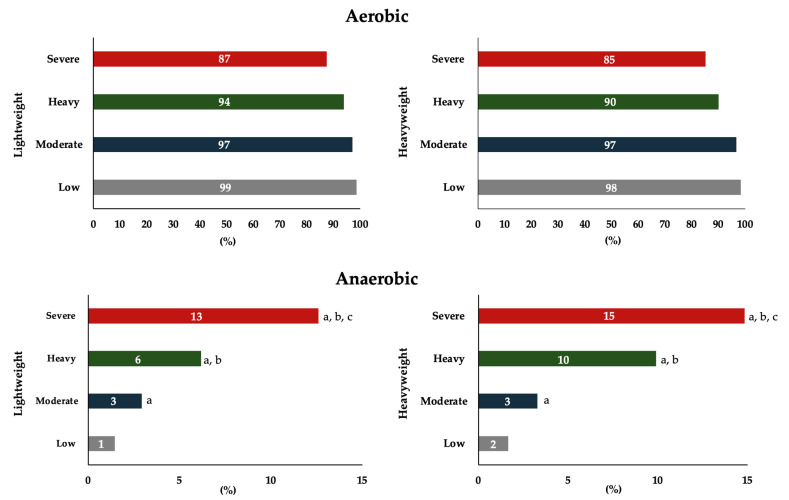
Relative aerobic and anaerobic energy contributions across four exercise intensity domains in lightweight and heavyweight rowers. ^a,b,c^: Different from low, moderate and heavy intensity domains within the same category (*p* ≤ 0.05).

Power output, cardiorespiratory variables and blood lactate concentrations increased progressively across exercise intensities, confirming the protocol’s effectiveness in eliciting graded physiological responses (Table 2). The comparison between lightweight and heavyweight rowers revealed a higher anaerobic energy output in the heavyweight group within the severe intensity domain (*p* = 0.02, *d* = 0.99), while no differences were observed for the remaining physiological variables between weight categories. Metabolic energy analysis showed a predominant reliance on aerobic metabolism across all intensities, particularly in the low and moderate domains (>85%), with anaerobic contribution becoming more pronounced in the heavy and severe domains (Figure 1). No differences were observed between lightweight and heavyweight rowers in the relative contributions of the metabolism system.

**Figure 2:**
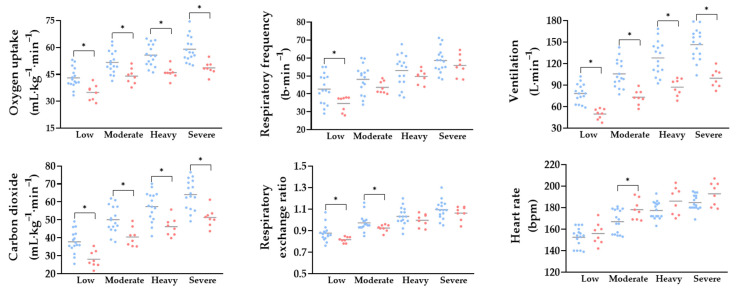
Cardiorespiratory variables assessed during rowing at low, moderate, heavy and severe exercise intensities in males (blue) and females (red). Data are presented as individual and mean values. *: Difference between sexes at the same intensity level (*p* ≤ 0.05).

**Figure 3:**
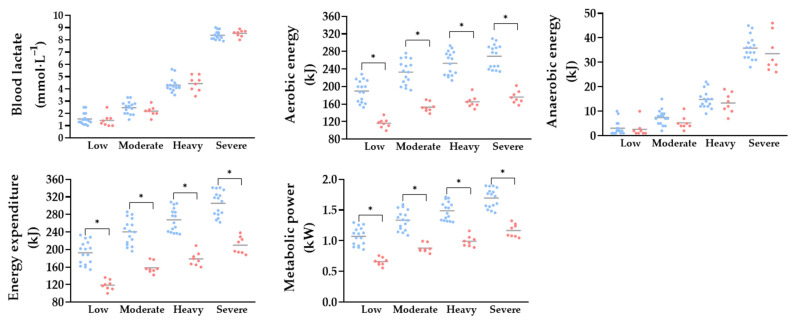
Metabolic variables assessed during rowing at low, moderate, heavy and severe exercise intensities in males (blue) and females (red). Data are presented as individual and mean values. *: Difference between sexes at the same intensity level (*p* ≤ 0.05).

Oxygen uptake (from ~43–59 vs. from ~34–48 mL·kg^−1^·min ^−1^), ventilation (from ~78–146 vs. from ~49–99 L∙min^–1^) and carbon dioxide production (from ~37–64 vs. from ~28–51 mL·kg^−1^·min^−1^) were higher in males than in females across all intensity domains, while respiratory frequency (~42 vs. ~34 b∙min^–1^) was higher in males only at low intensity (*p* < 0.05, *d* = 1.2). The respiratory exchange ratio was also higher in males compared with females at low and moderate (from ~0.87–0.97 vs. from ~0.81–0.92) intensities (*p* = 0.009 and 0.04, *d* = 1.1 and 0.9, respectively) with no differences at higher intensities. Heart rate increased in both sexes, with higher values in females than in males at moderate (~170 vs. ~178 bpm) intensity (*p* = 0.02, *d* = 1.1) (Figure 2). Aerobic contribution (from ~190–269 vs. from ~116–176 kJ) (*p* = 0.001, *d* = 3.85, 3.74, 4.12 and 4.30 for low, moderate, heavy and severe, respectively), energy expenditure (from ~193–305 vs. from ~119–209 kJ) (*p* = 0.001, d = 5.21, 0.80, 3.98 and 3.93 30 for low, moderate, heavy and severe, respectively) and metabolic power (from ~1.1–1.7 vs. from ~0.7–1.0 kJ) increased with exercise intensity (*p* = 0.001, *d* = 3.89, 2.91, 3.97 and 4.15 for low, moderate, heavy and severe, respectively), with males presenting higher values across all domains. No differences were observed between sexes in blood lactate concentrations and anaerobic contribution (Figure 3).

## 4. Discussion

This study compared physiological responses across four intensity domains in trained rowers, considering weight category and sex. We hypothesized that heavyweights would show higher cardiorespiratory and anaerobic outputs than lightweights and that males would demonstrate higher metabolic power and oxygen transport. Both hypotheses were partially confirmed: heavyweights had greater absolute outputs but similar relative responses, while males showed higher oxygen uptake and energy production yet comparable lactate levels and bioenergetic patterns, suggesting convergent internal regulation across groups.

The current results highlight the relevance of domain-based physiological profiling for characterizing the responses to incremental rowing at different exercise intensities. Heavyweight rowers exhibited greater anaerobic energy contribution in the severe intensity domain, which may be attributed to their higher muscle mass and total force-producing capacity [21,33]. In contrast, lightweight rowers appeared to sustain a comparable balance between performance and metabolic demand, suggesting a potential adaptive strategy to maintain relative economy. Male rowers showed higher values in oxygen transport and ventilatory variables across domains, particularly from the moderate to severe intensities, consistent with known sex-based differences in aerobic function [24,34]. These results partially confirm our initial hypotheses, with sex-related differences clearly observed, while differences between weight categories were limited to anaerobic energy contribution.

Despite these distinctions, blood lactate concentrations and anaerobic contributions were similar between sexes, suggesting equivalent engagement of glycolytic metabolism under maximal demand. This convergence may stem from shared recruitment of type II muscle fibers during high-intensity exertion, alongside similar training status among participants. Additionally, females may offset lower glycolytic capacity through enhanced metabolic efficiency and greater reliance on oxidative pathways, balancing lactate production under maximal load [27,34,35]. The current results diverge from the literature, suggesting differences between sexes, although further research is necessary to investigate the underlying causes of these differences, whether physiological or training-related [24,36,37]. For example, Cardoso et al. [24] found higher peak blood lactate concentrations in female rowers after an incremental exercise protocol. In contrast, Hill and Smith [36] and Held et al. [37] observed greater anaerobic performance in male athletes during short sprint efforts, including higher blood lactate accumulation, greater power output, and increased estimated anaerobic capacity. These findings suggest that while weight category and sex modulate absolute performance capacity, the internal physiological organization of effort may converge under standardized conditions. Such evidence reinforces the importance of using domain-specific assessments to guide training prescription, ensuring that load distribution reflects both individual and categorical physiological profiles [38].

Although lightweight and heavyweight rowers showed similar relative values for oxygen uptake, ventilation and heart rate, the heavyweight rowers produced more absolute energy and power at higher intensities, suggesting a distinct physiological demand associated with greater body mass [21,33]. This finding may be attributed to their greater muscle mass and force-generating capacity, which place greater demands on ventilatory and cardiovascular systems during intense exercise [33]. In contrast, lightweight rowers showed similar relative cardiorespiratory function, suggesting that they can sustain adequate physiological responses with lower energy cost. This result may be related to their higher power-to-body mass ratio, which improves muscle oxygenation, reducing metabolic strain during submaximal efforts; and to their technical proficiency [39,40]. Heavyweight rowers perform with greater absolute load, particularly on a rowing ergometer, while lightweight rowers reduce its difference on water [41].

The bioenergetic analysis demonstrated that aerobic metabolism was predominant at all exercise intensities, whereas anaerobic contribution progressively increased in the higher-intensity exertions regardless of weight category [3,4]. This response suggests a potential limitation in the rate of oxidative phosphorylation, necessitating compensatory activation of glycolytic pathways to meet the elevated energy demand. In contrast, lightweight rowers sustained a more stable predominance of aerobic metabolism, thereby exhibiting a higher activation of oxidative pathways. This may be attributed to their lower body mass; it is also plausible that enhanced oxygen diffusion capacity plays a contributory role [42]. This metabolic efficiency in lightweight rowers may also reflect superior technical proficiency and neuromuscular coordination, contributing to reduced physiological strain during effort. Furthermore, higher mitochondrial enzyme activity appears to support this oxidative dominance, facilitating sustained energy production under demanding physiological conditions [4,43]. Lightweight rowers accumulated less lactate for the same external workload, implying a lower metabolic cost and improved tolerance to high-intensity effort [44,45].

The analysis of cardiorespiratory variables revealed differences between sexes across all intensity domains, with males exhibiting higher values of oxygen uptake, ventilation and carbon dioxide production [6,46]. This functional advantage may be explained by morphological and hematological characteristics, particularly greater stroke volume, higher hemoglobin concentration and increased active muscle mass. These factors enhance the capacity for oxygen transport and diffusion, resulting in superior absolute aerobic performance, particularly at higher intensities. In contrast, females demonstrated higher heart rate values in the moderate domain, possibly reflecting an autonomic adjustment compensating for lower stroke volume [47]. Conversely, males exhibited higher respiratory frequency only at lower intensities, suggesting distinct ventilatory strategies depending on exercise domain. It is also important to note that the respiratory exchange ratio values were similar between sexes, indicating proportional substrate utilization despite differences in absolute workload. This metabolic convergence suggests that, under standardized progressive exercise conditions, both sexes rely equivalently on oxidative and glycolytic pathways.

Males exhibited higher values of aerobic energy, metabolic power and total energy expenditure across all intensity domains, with differences becoming progressively more pronounced as exercise intensity increased [24,48]. However, blood lactate levels remained similar between sexes, indicating that despite greater total energy production, men did not rely proportionally more on glycolytic pathways [47]. The progression of bioenergetic indicators followed a parallel pattern in both sexes, suggesting a similar internal metabolic organization in response to increasing intensity [48]. Nonetheless, males showed higher oxygen uptake, minute ventilation and carbon dioxide production, reflecting greater peripheral metabolic demand and a more pronounced ventilatory compensatory response [6,49]. These responses are associated with local muscular effects, such as increased oxygen extraction and metabolite accumulation and greater reliance on anaerobic glycolysis. In addition, males possess a higher capacity for ATP synthesis through both aerobic and anaerobic pathways, supporting greater overall energy production and enabling the maintenance of higher work rates [34,36]. Collectively, these factors contribute to the observed differences in bioenergetic response and emphasize the importance of considering sex-specific physiological traits in performance analysis.

### Limitations, Future Directions and Practical Applications 

Although the current study provides a relevant contribution to the understanding of physiological responses across different intensity domains between rowers of different weight categories and between sexes, several limitations should be acknowledged. Firstly, the relatively small sample size, particularly within the female group, may constrain the generalizability of the findings. Secondly, data collection was conducted in a laboratory setting using a Concept2 ergometer, which may not accurately replicate the physiological demands experienced in real competitive environments (particularly in on-water conditions). In addition, the estimation of energy system contribution was based on indirect methods, which may introduce a margin of error associated with the interpretations made. Furthermore, the cross-sectional nature of the study limits the ability to establish causal inferences and track training-induced physiological adaptations over time. Lastly, future research should incorporate indicators of biomechanical efficiency, as well as longitudinal designs that track training responses across different phases of the competitive season. It is also advisable to ensure greater sample diversity, with particular emphasis on increasing female representation, to deepen the understanding of physiological adaptations specific to different rowing profiles.

The obtained results have relevant practical implications for assessment, training prescription and load monitoring in rowers, enabling more targeted interventions according to weight category and sex by understanding how lightweight and heavyweight rowers differ in energy system contributions can support tailored pacing profiles that prevent early fatigue. In addition, sex-based differences in ventilatory and metabolic demands suggest the need for distinct recovery protocols post-race, including adjusted cooldown duration, nutritional support and monitoring of physiological recovery markers. The use of physiological domains defined by variables such as oxygen uptake, heart rate and blood lactate concentration allow for individualized training zone prescription, optimizing stimulus application while minimizing the risk of accumulated fatigue. For heavyweight rowers, greater emphasis is recommended on sessions that enhance anaerobic tolerance, particularly through high-intensity interval blocks with controlled recovery periods, aiming to improve buffering capacity and glycolytic pathway utilization. Conversely, lightweight rowers should prioritize the development of aerobic efficiency, including prolonged moderate intensity sessions and extensive work at the anaerobic threshold, given their relative advantage in exercise economy. Regarding sex-specific approaches, men may benefit from higher training loads in more intense domains, tailored to their higher absolute physiological capacity, while females hold emphasized volume accumulation at submaximal intensities, promoting metabolic stability and oxidative efficiency. Systematic use of heart rate monitors and capillary lactate measurements is strongly recommended for supporting immediate load adjustments and facilitating early detection of overreaching or undertraining signs.

## 5. Conclusions

This study demonstrated that domain-based physiological profiling is an effective approach for distinguishing the internal responses of rowers across different weight categories and sexes. Heavyweight rowers exhibited greater absolute energy output and anaerobic contribution in higher-intensity domains, reflecting their greater muscle mass and force-producing potential. In contrast, lightweight rowers showed similar relative cardiorespiratory responses, suggesting greater metabolic economy under equivalent workload conditions. Male rowers presented higher values of oxygen uptake, ventilation, carbon dioxide production and energy expenditure across all domains, which were consistent with known sex-based differences in oxygen transport and substrate utilization. Despite these differences in absolute values, the relative bioenergetic contributions and lactate accumulation patterns were comparable between groups, indicating a convergent internal regulation of metabolic pathways. These findings underscore the need for individualized training prescriptions that reflect the unique physiological characteristics associated with sex and body mass.

## Figures and Tables

**Table 1:** Demographic and anthropometric characteristics of participants and the distribution within each group (lightweight and heavyweight).

Variables	Lightweight *n* = 12	Heavyweight *n* = 12	Males *n* = 16	Females *n* = 8 *
Age (years)	24.83 ± 9.82	23.92 ± 8.04	26.40 ± 9.45	20.13 ± 6.64
Height (cm)	173.94 ± 6.92	177.53 ± 9.95	179.30 ± 7.48	168.53 ± 6.39
Body Mass (kg)	75.99 ± 21.26	80.41 ± 11.34	80.39 ± 9.19	74.43 ± 27.07
Lean Mass (%)	61.63 ± 20.28	57.63 ± 15.70	49.12 ± 14.28	39.09 ± 11.57
Fat Mass (%)	15.37 ± 8.62	14.80 ± 5.36	10.91 ± 2.97	22.87 ± 6.14
Experience (years)	9.14 ± 3.24	9.22 ± 2.44	10.43 ± 4.35	10.75 ± 0.46

* Four lightweights and four heavyweights.

**Table 2:** Performance and physiological demands at low, moderate, heavy and severe intensities among lightweight and heavyweight rowers.

Variables	Low		Moderate		Heavy		Severe	
	Lightweight	Heavyweight	Lightweight	Heavyweight	Lightweight	Heavyweight	Lightweight	Heavyweight
Power (W)	184.17 ± 44.81 ^†^	183.33 ± 54.33 ^†^	238.33 ± 52.54 ^a,†^	240.01 ± 58.31 ^a,†^	269.17 ± 55.34 ^a,b,†^	277.50 ± 61.07 ^a,b,†^	297.50 ± 57.54 ^a,b,c,†^	305.83 ± 63.45 ^a,b,c,†^
Oxygen uptake (mL∙kg^−1^∙min^–1^)	42.20 ± 6.21 ^†^	38.50 ± 6.43 ^†^	51.42 ± 6.95 ^a,†^	46.82 ± 5.92 ^a,†^	54.78 ± 7.17 ^a,b,†^	50.17 ± 6.55 ^a,b,†^	58.42 ± 8.62 ^a,b,c,†^	52.67 ± 6.07 ^a,b,c,†^
Respiratory frequency (b∙min^–1^)	42.35 ± 12.95 ^§^	37.20 ± 9.58 ^§^	44.97 ± 10.86 ^§^	45.56 ± 8.47 ^a,§^	51.31 ± 9.84 ^a,b,§^	50.62 ± 9.30 ^a,b,§^	57.41 ± 11.38 ^a,b,c,§^	56.68 ± 9.15 ^a,b,§^
Ventilation (L∙min^–1^)	67.87 ± 16.98 ^†^	71.67 ± 19.29 ^†^	90.99 ± 21.14 ^a,†^	100.60 ± 24.75 ^a,†^	109.02 ± 24.56 ^a,b,†^	121.80 ± 30.75 ^a,b,†^	125.62 ± 27.93 ^a,b,c,†^	137.64 ± 30.59 ^a,b,c,†^
Carbon dioxide (mL∙kg^−1^∙min^–1^)	36.30 ± 7.64 ^§^	32.78 ± 7.04 ^§^	49.34 ± 8.39 ^a,§^	44.51 ± 7.40 ^a,§^	56.16 ± 9.28 ^a,b,§^	51.17 ± 8.48 ^a,b,§^	63.30 ± 10.07 ^a,b,c,§^	56.35 ± 7.85 ^a,b,c,§^
Respiratory exchange ratio	0.86 ± 0.09 ^†^	0.84 ± 0.06 ^†^	0.96 ± 0.09 ^a,†^	0.94 ± 0.07 ^a,†^	1.03 ± 0.08 ^a,b,†^	1.01 ± 0.08 ^a,b,†^	1.10 ± 0.09 ^a,b,c,†^	1.07 ± 0.08 ^a,b,c,†^
Heart rate (bpm)	154 ± 12 ^§^	149 ± 12 ^§^	171 ± 17 ^a,§^	168 ± 13 ^a,§^	178 ± 21 ^a,b,§^	176 ± 14 ^a,b,§^	184 ± 18 ^a,b,c,§^	184 ± 13 ^a,b,c,§^
Blood lactate (mmol∙L^–1^)	1.55 ± 0.77 ^#^	1.58 ± 0.51 ^#^	2.41 ± 0.71 ^a,#^	2.43 ± 0.43 ^a, #^	4.23 ± 1.19 ^a,b,#^	6.93 ± 8.12 ^a,b,c,#^	8.58 ± 1.86 ^a,b,c,#^	8.27 ± 3.42 ^a,b,c,#^
Aerobic energy (kJ)	163.24 ± 38.82 ^†^	170.34 ± 43.73 ^†^	203.08 ± 43.31 ^a,†^	212.13 ± 46.70 ^a,†^	218.15 ± 48.87 ^a,b,†^	229.03 ± 50.78 ^a,b,†^	234.15 ± 52.93 ^a,b,c,†^	241.59 ± 50.31 ^a,b,c,†^
Anaerobic energy (kJ)	2.25 ± 2.83 ^#^	3.16 ± 3.02 ^#^	6.08 ± 2.91 ^a,#^	7.36 ± 2.98 ^a,#^	13.86 ± 4.48 ^a,b,#^	29.64 ± 39.40 ^a,b,#^	32.54 ± 5.92 ^a,b,c,#^	41.25 ± 10.39 ^a,b,c,^*^,#^
Energy expenditure (kJ)	165.50 ± 37.01 ^†^	168.74 ± 43.64 ^†^	209.17 ± 42.27 ^†^	213.38 ± 46.92 ^a,†^	232.02 ± 44.96	254.16 ± 73.07 ^a,b,†^	266.69 ± 51.59	277.52 ± 51.23 ^a,b,c,†^
Metabolic power (kW)	0.92 ± 0.21 ^†^	0.96 ± 0.25 ^†^	1.16 ± 0.25 ^a,†^	1.22 ± 0.27 ^a,†^	1.19 ± 0.26 ^a,b,†^	1.44 ± 0.42 ^a,b,†^	1.48 ± 0.30 ^a,b,c,†^	1.57 ± 0.30 ^a,b,c,†^

Data are presented as the mean ± standard deviation. ^a,b,c,^*: Differences from low, moderate and heavy exercise intensities and from lightweight rowers (respectively, *p* ≤ 0.05). Eta square: ^†^: [0.25–0.50]; ^§^: [0.51–0.75]; ^#^: [0.76–1].

## Data Availability

All data is contained within the article.
